# *Undaria pinnatifida* ameliorates nasal inflammation by inhibiting eosinophil and mast cell activation and modulating the NF‐κB/MAPKs signaling pathway

**DOI:** 10.1002/iid3.1215

**Published:** 2024-03-15

**Authors:** Zhen Nan Yu, Yan Jing Fan, Thi Van Nguyen, Chun Hua Piao, Byung‐Hoo Lee, So‐Young Lee, Hee Soon Shin, Chang Ho Song, Ok Hee Chai

**Affiliations:** ^1^ Department of Anatomy Jeonbuk National University Medical School Jeonju South Korea; ^2^ Department of Basic Medicine, School of Medicine Liaocheng University Liaocheng Shandong China; ^3^ Department of Pulmonary and Critical Care Medicine Yantai Yuhuangding Hospital Yantai China; ^4^ Department of Food Science and Biotechnology Gachon University Seongnam South Korea; ^5^ Division of Food Functionality Research Korea Food Research Institute Wanju South Korea; ^6^ Division of Food Biotechnology Program Korea University of Science and Technology Daejeon South Korea; ^7^ Institute for Medical Sciences Jeonbuk National University Jeonju South Korea

**Keywords:** allergic rhinitis, anti‐inflammation, eosinophils, mast cells, NF‐κB/MAPKs, *Undaria pinnatifida*

## Abstract

**Background:**

Allergic rhinitis (AR) is the most prevalent form of atopic disease. *Undaria pinnatifida* has potent antioxidative, antidiabetic, and anti‐inflammatory properties.

**Aims:**

We investigated the immunomodulatory effect of *Undaria pinnatifida* extract (UPE) on allergic inflammation in an AR mouse model.

**Materials & Methods:**

Mice were sensitized and intranasally challenged with ovalbumin (OVA), and the Th1/Th2 and Th17/Treg‐related cytokines and histopathology were exanimated after UPE treatments. Enzyme‐linked immunosorbent assay was performed using serum samples and NALF to detect OVA‐specific immunoglobulins and inflammatory cytokines. Mitogen‐activated protein kinases (MAPKs) were measured by western blotting analysis, and an in vitro study measured mast cell activation induced by compound 48/80.

**Results:**

After UPE treatment, nasal and lung allergy symptoms, nasal mucosal swelling, and goblet cell hyperplasia were ameliorated. Oral UPE regulated the balance of Th1/Th2 and Th17/Treg cell differentiation in AR mice in a dose‐dependent manner. In addition, UPE attenuated the migration of eosinophils and mast cells to the nasal mucosa by suppressing nuclear factor kappa B (NF‐κB)/MAPKs. The levels of anti‐OVA IgE and IgG_1_ were also decreased.

**Discussion:**

UPE inhibited inflammation by regulating the NF‐κB/MAPKs signaling pathway and supressing the activation of critical immune cells such as eosinophils and mast cells.

**Conclusion:**

UPE may have therapeutic potential for AR.

## Graphical abstract

Allergic rhinitis (AR) is the most prevalent form of atopic disease. By modulating the NF‐κB/MAPKs signaling pathway and inhibiting the activation of critical immune cells such as eosinophils and mast cells, Undaria pinnatifida extract may have therapeutic potential for AR.

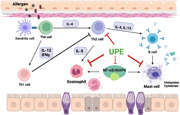

**Abbreviations**
AR: allergic rhinitis
Ig: immunoglobulin
IL: interleukin
INF‐γ: interferon‐gamma
MAPK: mitogen‐activated protein kinases
NALF: nasal lavage fluid
NF‐κB: nuclear factor‐kappa B
OVA: ovalbumin
Th: T helper
TNF‐α: tumor necrosis factor‐alpha
Treg: T regulatory
UPE: *Undaria pinnatifida* extract

## 1 INTRODUCTION

Allergic rhinitis (AR) is the most observed manifestation of atopic illness. The prevalence of AR among adults in the United States and Europe ranges from 20% to 30%, with the possibility of a higher occurrence in children.^1^ The starting symptoms include nasal congestion, sneezing, and nasal itching; patients with AR experience a decrease in productivity.^2^

AR is an allergic immune response triggered by an inhaled allergen; the immune response reacts with the allergen by activating dendritic cells and T lymphocytes.^3^ CD4+ T‐cells play a key role in the initiation and orchestration of the allergic immune response through the secretion of cytokines such as interleukin‐4 (IL‐4), IL‐5, IL‐10, and IL‐13.^4^ IL‐4 is a pivotal cytokine that drives sensitization to allergens by inducing the immunoglobulin E (IgE) class switch in B lymphocytes.^5^ When allergens encounter the nasal mucosa of individuals who are hypersensitive, they attach to allergen‐specific IgE located on mast cells. This interaction triggers the immediate release of prepared mediators, which subsequently induce early nasal symptoms.^6^ Histamine and tumor necrosis factor‐alpha (TNF‐α) released from mast cells can promote inflammatory cell (e.g., eosinophil) influx by stimulating the expression of adhesion factors on the endothelial cells.^7^ The inflammatory response in AR is due to the activation of the major transcription factor nuclear factor kappa B (NF‐κB).^8^ In addition, mitogen‐activated protein kinases (MAPKs) regulate the expression of Th2 cytokines and influence the differentiation of inflammatory cells such as Th2 and eosinophils.^9^ Many signaling pathways in eosinophils are activated by the Th2 cytokine IL‐5, including MAPKs/NF‐κB, and the combination of these kinases and transcription factors stimulates eosinophil differentiation, survival, degranulation, and adhesion.^10^ This is characteristic of a late allergic reaction, where the nasal congestion is the main symptom.^11^

Oral leukotriene receptor antagonists are often prescribed for AR patients, and the patients with episodic AR are treated using intranasal antihistamines or glucocorticoids.^12^ However, excessive use of glucocorticoids can be detrimental to the health of patients.^13^ In recent years, several promising novel therapies may offer more effective or safe treatment options for patients with AR.^14^ *Undaria pinnatifida* (*U. pinnatifida*) is a brown alga of the class Phaeophyta, order Laminariales, family Alariaceae, and genus Undaria.^15^ It is characterized as a temperate annual species, and the general morphology of *U. pinnatifida* is characterized by a yellowish‐brown hue, possessing a smooth texture. It has a prominent thallus structure composed of a substantial algal body, featuring wide and flat fronds. Notably, there is a conspicuous midrib bulge, while the lateral margins of the fronds appear relatively thin.^16^ *U. pinnatifida* has been historically incorporated into the traditional diet of East Asia and is commonly referred to as a “longevity food” or “sea of vegetables” in various western countries.^17^ According to the authoritative ancient texts of traditional Chinese medicine, such as the “Compendium of Materia Medica” from the Ming Dynasty, *U. pinnatifida* is characterized by a bitter and salty taste, a cold nature, and inherent non‐toxicity. It is believed to possess the therapeutic properties of promoting diuresis to alleviate edema, facilitating the softening of hardened tissues, and reducing inflammation.^18^ Within the category of brown seaweeds. *U. pinnatifida* stands out as a notable reservoir of many essential nutrients. This species is characterized by its high protein content and dietary fiber, as well as significant amounts of calcium, sodium, potassium, iron, magnesium, vitamin B, vitamin A, and antioxidants.^19^ *U. pinnatifida* is well known for containing phenolic compounds^20^ with antioxidant,^21^ anti‐inflammatory,^22^ antidiabetic,^23^ and antibacterial^24^ activities. Furthermore, our quantitative analysis showed that galactans was the most abundant compound in *U. pinnatifida*. The galactose component of *U. pinnatifida*, which mainly produces galactose‐fucoidan (galactofucans), has anti‐inflammatory and anti‐tumor effects.^25^ Meanwhile, galactose from fucoidan showed potent antiviral activity against herpes simplex virus type 1 (HSV‐1), HSV‐2 and human cytomegalovirus.^26^ However, few studies have been published on the pharmacological activity of *U. pinnatifida*. Therefore, to the best of our knowledge, this study is the first to investigate the protective effect of UPE on an ovalbumin (OVA)‐induced AR mouse model and to explore its possible mechanisms. Herein, we explored the allergic inflammatory response of UPE to OVA‐induced AR mice. In addition, we investigated the specific mechanism by which UPE ameliorates AR by modulating the MAPK/NF‐κB pathway.

## 2 MATERIALS AND METHODS

### 2.1 Animals

Nearly 6‐week‐old male BALB/c mice (Daejeon, Korea) and 8‐month‐old male Sprague‐Dawley rats (Daejeon, Korea) were used as experimental animals. The mice and rats were raised in the laboratory for 1 week at 23–25°C, relative humidity of 10%–50%, and 12/12 h light/dark cycles. All animal experiments conducted in this study adhered to the rules for animal care and use as outlined by the Jeonbuk National University Laboratory Animal Center (JBNU 2021‐0115).

### 2.2 Preparation of UPE

**Figure 1:**
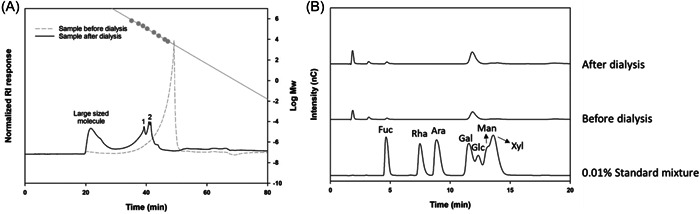
(A) The before‐dialysis sample contained a higher amount of molecules smaller than the standard substance (6300 Da), which were removed after dialysis. And the after‐dialysis sample was confirmed the presence of very large molecular weight substances (in the 20‐min range) and substances primarily between 63,000 and 160,000 Da (in the 1–2 peak region). (B) The main sugars comprising both the pre‐ and post‐dialysis samples were identified as galactose and a small amount of fucose, with no significant differences in the types of sugars present before and after dialysis. It was observed that the quantity of galactose increased after dialysis and acid hydrolysis.

Dried *U. pinnatifida* (seaweed) was acquired from Gijang, South Korea. To obtain UPE, these were extracted (70% ethanol at 50°C for 6 h), concentrated (at 50°C using a rotary evaporator BÜCHI Labortechnik AG), and freeze‐dried (freeze dryer OPERON). The obtained powder was stored at −80°C. And we analyzed various components using UPLC‐Q‐TOF MS. However, due to the high sugar content characteristic of *Undaria Pinnatifida*, it was necessary first to analyze the sugar content and then proceed with UPLC‐Q‐TOF MS after removing the sugars. We anticipate that the functional components of the *Undaria Pinnatifida* extract are polysaccharides and phytochemicals, and the result (Figure 1B) has demonstrated the analysis of polysaccharides. By comparing the molecular size before and after dialysis, we found that before dialysis, molecules smaller than 6300 Da were prevalent, but after dialysis, large size molecules predominantly between 63,000 and 160,000 Da were present (Figure 1A). Additionally, the analysis of the sugars before and after dialysis showed an increase in the proportion of galactose, leading us to confirm that the functional components of this *Undaria Pinnatifida* extract is galactans (Figure 1B).

### 2.3 AR model establishment and treatment

**Figure 2:**
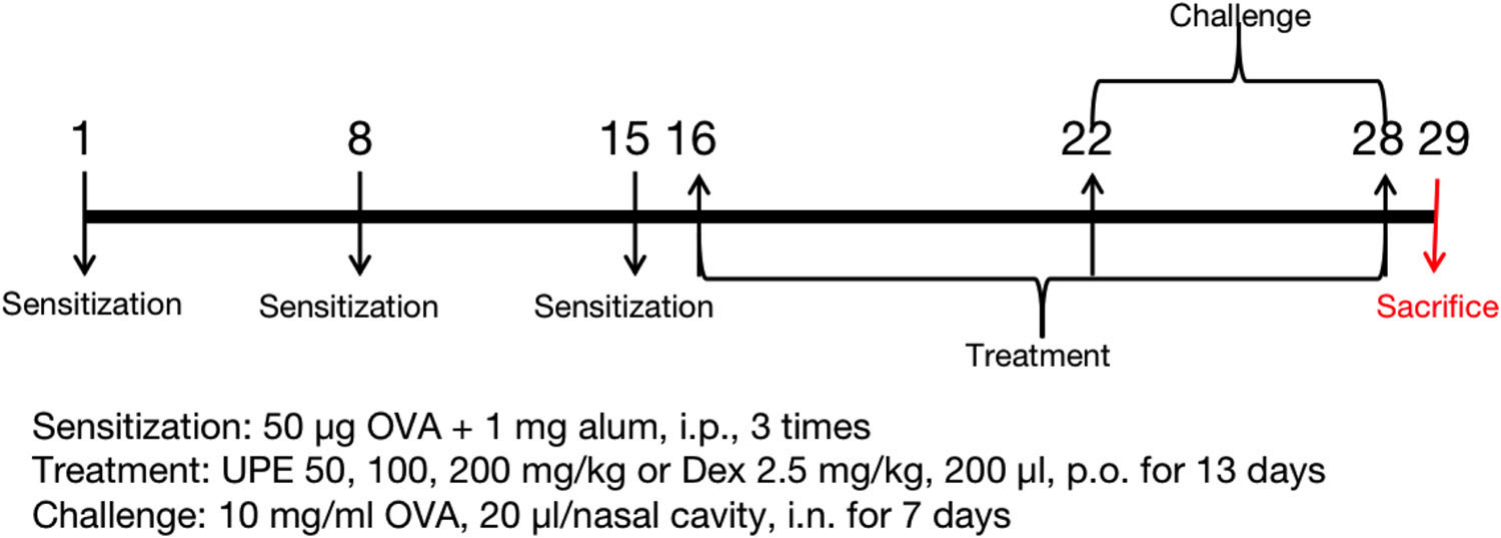
Establishment of an AR mouse model and treatment with UPE. Mice were sensitized on days 1, 8, and 15 and OVA‐challenged on days 22–28. Mice in the UPE or Dex groups were administered orally once daily at 50, 100, 200 mg/kg UPE or 2.5 mg/kg Dex for 13 days, respectively. AR, allergic rhinitis; UPE, *Undaria pinnatifida* extract.

BALB/c mice were randomly divided into the following six (*n* = 6) groups: control; OVA; UPE 50, 100, and 200 mg/kg; Dexamethasone (Dex) 2.5 mg/kg. The establishment of the OVA‐induced AR mouse model and the treatment protocol was shown in the Figure 2. In this study, AR was generated in mice through the administration of OVA. The mice were made more sensitive on days 1, 8, and 15 by injecting them with 200 μL of saline solution that had 50 μg of OVA (Grade V, Sigma) attached to 1 mg of aluminum hydroxide (Thermo Scientific). One week after the last sensitization, mice were challenged with 10 mg/mL of OVA through the nose from days 22 to 28. The administration involved the application of 20 μL per nasal cavity. Mice were treated with oral UPE and Dex once daily from days 16 to 28 (1 h before intranasal OVA challenge on days 22–28). Mice in the OVA and control groups received a sham saline volume. Mice were killed 24 h after the last OVA challenge.^27^

### 2.4 Observation of nasal symptoms

Mice were intranasally challenged with 10 mg/mL OVA (20 μL per nasal cavity) for 7 consecutive days from days 22 to 28. After the last challenge, the sneezing and nasal rubbing behaviors were video recorded during a 15‐min period and then counted by blinded observers.

### 2.5 Nasal lavage fluid (NALF) collection and cell count

After sacrificing the mice, the trachea was opened, and 1 mL of sterile saline solution was gently pumped into the nasal cavity using an 18‐gauge catheter. NALF was collected from the anterior nasal cavity after centrifugation at 10,000 rpm for 10 min at 4°C. The supernatant was then transferred to another clean tube and stored at −80°C to determine cytokine levels. The cell pellets were resuspended in a cold, sterile saline solution, and the quantification of the total cell count was performed using a hemocytometer. To perform differential cell counts, a volume of 150 μL NALF was subjected to centrifugation onto slides using a cell cytospin equipment (Centrifuge 5403, Eppendorf) at a speed of 1000 revolutions per minute for 10 min at 4°C. The relative numbers of different cell types were determined using the Diff‐Quik staining kit. Inflammatory cells were counted under a light microscope (Leica) at ×400.

### 2.6 Histopathological examination of nasal and lung tissues

After collecting NALF, the heads and lungs of the mice were removed and fixed in 10% paraformaldehyde solution for 3 days. The heads were decalcified in ethylenediaminetetraacetic acid for 7 days, and all specimens (except for the lungs) were embedded in paraffin. The tissue samples were sliced into sections that were 4 μm in thickness. These sections were then subjected to staining using hematoxylin and eosin (H&E, Sigma). This staining method was employed to observe the overall morphology of the tissue. Additionally, periodic acid‐Schiff (PAS, Sigma) was used to assess the proliferation of goblet cells. The nasal tissues were subjected to treatment with Giemsa and Toluidine Blue (Sigma) to evaluate the infiltration of eosinophils and mast cells. The quantification of eosinophils and mast cells was conducted in both the nasal septum and lateral processes. Epithelial damage analysis was performed using random selection of a light microscope at ×400 magnification.

### 2.7 Measurement of OVA‐specific immunoglobulins in serum

Blood was collected from the orbital venous plexus of anesthetized mice 24 h after the last OVA challenge. Samples were centrifuged (10,000 rpm, 10 min, 4°C) to collect serum and stored at −80°C until analysis. Enzyme‐linked immunosorbent assay (ELISA) was performed using serum samples to detect OVA‐specific IgE (BioLegend), IgG_1_ (Cayman), and IgG_2a_ (Ehondrex) according to the manufacturer's instructions.

### 2.8 Quantification of cytokines by ELISA

The supernatants collected from the NALF were used to quantify cytokine release. Cytokine quantification kits were employed for assessing cytokine concentrations, namely those of IL‐4, IL‐5, IL‐13, IFN‐γ, IL‐12, IL‐10, IL‐17, IL‐6, eotaxin, histamine (R&D Systems), NF‐κB p65 (Mybiosource), and phosphorylation of NF‐κB p65 (p‐NF‐κB p65, Cell Signaling Technology); all assays were performed in duplicate according to the manufacturer's instructions. The optical density (OD) was measured at 450 nm in a 96‐well plate using an ELISA reader.

### 2.9 Western blotting

The lung tissue homogenates were centrifuged (10,000 rpm, 5 min, 4°C), and the supernatants were collected as total protein extracts. Protein concentrations were determined using the bicinchoninic acid method. Protein samples of equal quantities were subjected to separation using a 10% sodium dodecyl sulfate‐polyacrylamide gel electrophoresis technique, followed by transfer onto a polyvinylidene difluoride membrane. The membrane was blocked with 5% dried milk for 1 h at room temperature followed by overnight incubation (at 4°C and gentle shaking) with a monoclonal antibody against β‐actin (Cell Signaling Technology) and MAPKs (Cell Signaling Technology). The membrane was incubated with a secondary antibody (1:3000) for 90 min at room temperature followed by incubation with a horseradish peroxidase‐conjugated antibody (1:1000) for similar duration and condition. Immunoreactive bands were detected using a detection solution. The quantification of band intensity was performed using Image Lab version 4.0 software, developed by Bio Rad Laboratories. The protein β‐Actin was employed as a control for loading proteins.

### 2.10 In vitro study with compound 48/80‐induced mast cell activation

After anesthetizing the rats, 50 mL of saline was injected intraperitoneally, and the abdomen was gently kneaded for 90 s. The peritoneal cavity was carefully opened, and the fluid was aspirated using a Pasteur pipette. The peritoneal lavage fluid was collected and centrifuged (1000 rpm, 10 min, 4°C). The supernatant was discarded, and the cells were collected and re‐suspended in 7 mL of HEPES buffer. Rat peritoneal mast cells (RPMCs) were pre‐incubated with different concentrations (1, 10, and 100 mg/mL) of UPE for 10 min at 37°C, followed by incubation with compound 48/80 (C48/80; 5 μg/mL) for 15 min at 37°C. After incubation, RPMCs were placed on slides and observed under a high‐power microscope; the degranulation rate of RPMCs was counted under ×400 magnification.

### 2.11 Cell viability assay

The determination of cell viability was conducted via the MTT test. RPMCs were cultured in 96‐well plates with a cell density of 1 × 10^5^ cells/mL for an overnight incubation period. The cells were subjected to treatment with different concentrations of UPE for a duration of 4 h, following which MTT (0.5 mg/mL) was added, and the cells were incubated for an additional 4 h. The liquid portion was extracted, and a solution of hydrochloride‐isopropanol was introduced to the solid material and agitated until complete dissolution occurred. The OD was quantified at a wavelength of 570 nm.

### 2.12 Statistical analysis

GraphPad Prism software (v5.0) was used to analyze the results, while the statistical significance of differences among groups was analyzed using one‐way Analysis of variance followed by Tukey's test. Data were expressed as the standard error of the mean of independent experiments. Significance was considered at the 95% confidence level (*p* < .05).

## 3 RESULTS

### 3.1 UPE improved the symptoms of OVA‐induced AR

**Figure 3:**
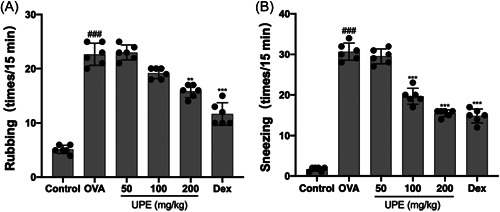
UPE suppressed the symptoms of OVA‐induced nasal allergy. (A) Rubbing scores. (B) Sneezing scores. Rubbing and sneezing events were counted within 15 min after the last OVA challenge. UPE reduced the frequency of rubbing and sneezing in the OVA‐induced AR mouse model. Data were obtained from the results of six separate experiments and displayed as mean ± *SD*. ANOVA test was applied to compare the differences among multiple groups, respectively. Significant differences at ###*p* < .001 compared with the control group. Significant differences at **p < .01, ***p < .001 compared with the OVA group. ANOVA, analysis of variance; OVA, ovalbumin; UPE, *Undaria pinnatifida* extract.

To assess the impact of UPE on initial allergic symptoms, the frequency of sneezing and rubbing was quantified 15 min after the most recent intranasal OVA challenge. The stroking and sneezing scores of the OVA‐induced AR group were significantly higher than those of the control group, according to the findings; however, significantly lowering was observed in the UPE and Dex groups compared with the OVA group (Figure 3A,B). These findings suggest that UPE treatment is effective in improving OVA‐induced allergic nasal symptoms.

### 3.2 UPE reduced the differential inflammatory cells infiltration in NALF

**Figure 4:**
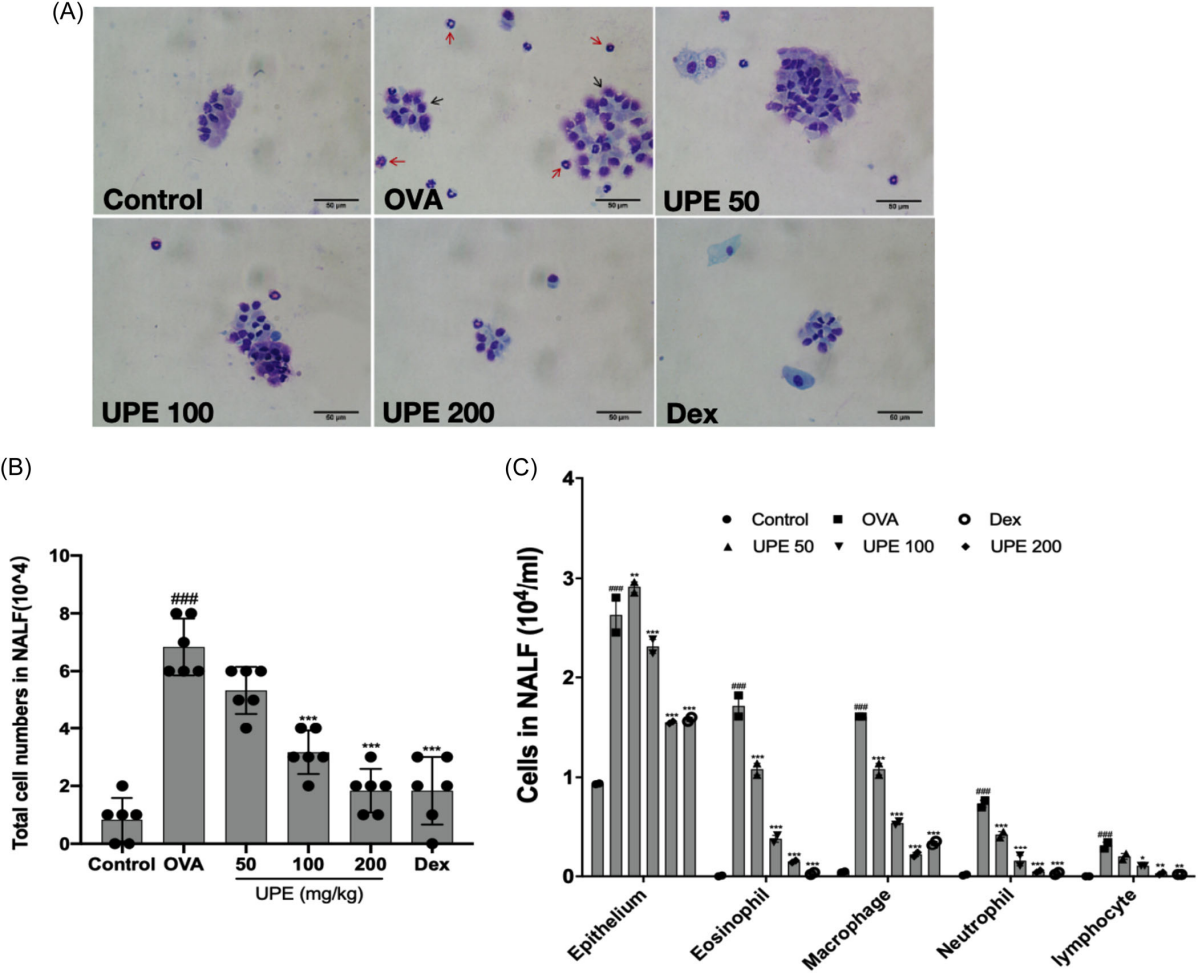
UPE decreased the infiltration of differential inflammatory cells in NALF in a dose‐dependent manner. (A) Differential cells were stained with Diff‐Quik (B) Total number of cells in NALF. Data were obtained from the results of six separate experiments and displayed as mean ± *SD*. (C) The number of differential cells in NALF, and the values represent the mean ± SEM (*n* = 2/group). NALF was collected immediately after sacrifice and cells were isolated by cytospin. Red and black arrows indicate eosinophils and lost epithelial cells, respectively. Scale bar 50 μm, ×400 magnification. Significant differences at ###*p* < .001 compared to the control group. Significant differences at *p < .05, **p < .01, ***p < .001 compared to the OVA group. ANOVA, analysis of variance; NALF, nasal lavage fluid; OVA, ovalbumin; SEM, standard error of the mean; UPE, *Undaria pinnatifida* extract.

Differential inflammatory cells in NALF were identified by Diff‐Quik staining. Eosinophils displayed two lobes of their nucleus and red‐stained granules in the cytoplasm; occasionally, the nucleus took the form of a ring. Neutrophils had two to five lobes in their nucleus and pink cytoplasm when they first emerged. Macrophages were huge, often bean‐shaped cells with a big, dark blue nucleus. Cilia were the defining feature of an epithelial cell, and its nucleus was situated on the side opposite the cilia.^28^ which showed that the epithelial cells and eosinophils were hyper‐expressed in the OVA group. In contrast, they were significantly reduced in UPE‐ and Dex‐treated mice (Figure 4A). The total number of cells and differential cells (including epithelial cells, eosinophils, neutrophils, lymphocytes, and macrophages) in the OVA group was significantly higher than of the control group (Figure 4B,C). In contrast, UPE at doses of 100 and 200 mg/kg markedly decreased the number of these inflammatory cells in NALF (Figure 4B,C). These results suggest that the UPE ameliorates the aggravation of nasal epithelial barrier disruption.

### 3.3 UPE improved the thickness and mucus secretion of nasal mucosa and inflammation of lung tissue

**Figure 5:**
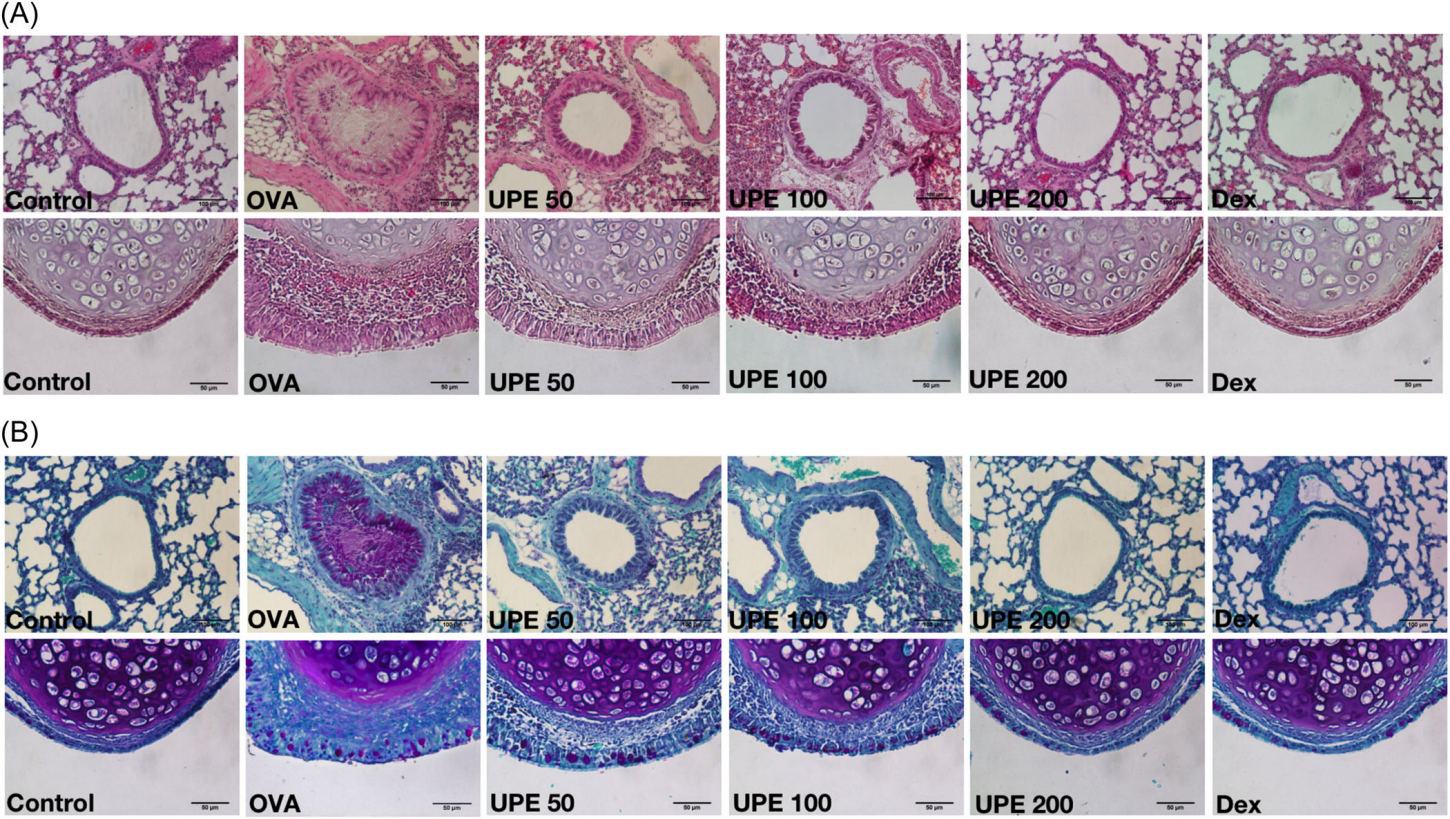
UPE improved the inflammation of nasal and lung tissues. (A) Histological features of the nasal mucosa and lungs were determined by H&E staining (B) Goblet cells were identified by PAS staining. UPE ameliorates pathological changes in nasal and lung tissue of mice. Scale bar 50 μm, ×400 magnification, 100 μm, ×200 magnification. H&E, hematoxylin and eosin; UPE, *Undaria pinnatifida* extract.

The general morphology of the nasal and respiratory tracts was assessed using H&E staining. Histological changes were observed in the OVA group with significant aggregation of sub‐epithelial inflammatory cells, resulting in a marked increase in mucosal thickness (Figure 5A). After UPE and Dex administration, few pathological changes in the nasal mucosa and lung tissue returned to normal. PAS staining showed mucus overproduction and goblet cell hyperplasia in the bronchi and nasal cavity in the OVA group compared with the control group (Figure 5B). The goblet cells in the OVA group carried an apparent purple mucus secretion that was alleviated in mice treated with high concentrations of UPE (200 mg/kg) and Dex groups, but not significantly improved at low UPE (50 mg/kg) concentration. Therefore, UPE administration showed a dose‐dependent protective effect on the nasal mucosa and lung tissues.

### 3.4 UPE modulated the OVA‐specific Igs in serum of OVA‐induced AR mice

**Figure 6:**
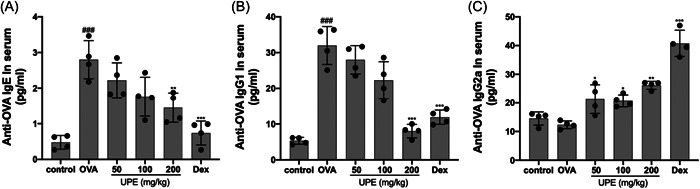
UPE modulated the levels of anti‐OVA antibodies in serum. Mice treated with UPE downregulated the levels of anti‐OVA IgE (A) and anti‐OVA IgG_1_ (B), while upregulated anti‐OVA IgG_2a_ (C). Data were obtained from the results of four separate experiments and displayed as mean ± *SD*. ANOVA test was applied to compare the differences among multiple groups, respectively. Significant differences at ###*p* < .001 compared to the control group. Significant differences at *p < .05, **p < .01, ***p < .001 compared with the OVA group. ANOVA, analysis of variance; IgE, immunoglobulin E; OVA, ovalbumin; UPE, *Undaria pinnatifida* extract.

The crucial involvement of antibodies in the immune system is widely recognized. Repeated exposures to allergens induce the development of IgE and IgG_1_, hence augmenting the immunological response. The creation of IgG_2a_ is contingent upon the presence of Th1 cells, which play a regulatory role in the activity of IgE and IgG_1_.^29^ Compared with the control group, anti‐OVA IgE (Figure 6A) and IgG_1_ (Figure 6B) were markedly elevated, and anti‐OVA IgG_2a_ (Figure 6C) was downregulated in the OVA group. In contrast, UPE administration resulted in dose‐dependent lowering of anti‐OVA IgE and IgG_1_ levels. In contrast, anti‐OVA IgG_2a_ levels were higher in the UPE‐treated group than in the OVA group, and the same thing happened in mice that were given Dex. In summary, the findings of this study indicate that UPE has the potential to enhance anti‐allergic activity through its ability to suppress the synthesis of allergy mediators.

### 3.5 UPE regulated the balance of Th1/Th2/Th17/Treg‐related cytokines in NALF

**Figure 7:**
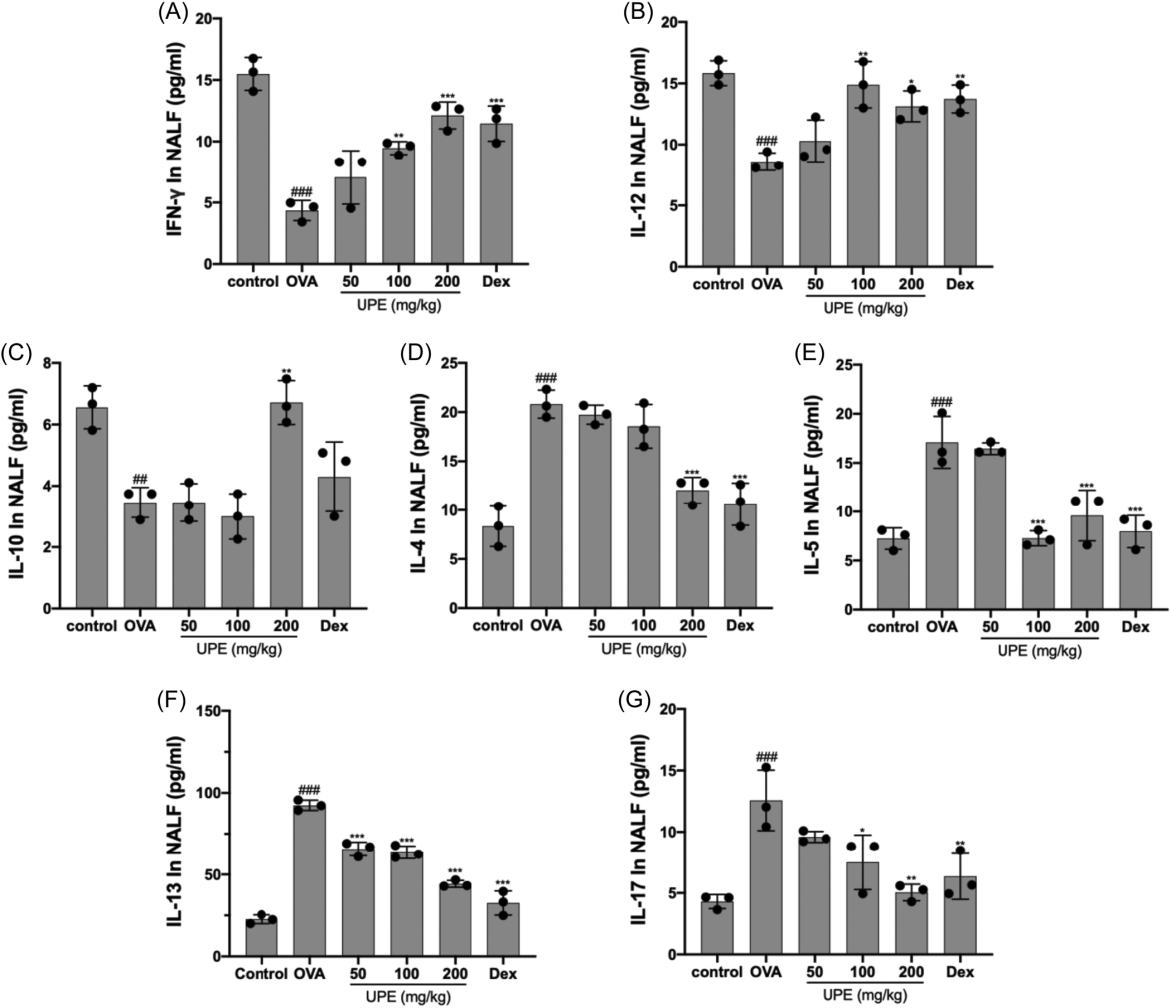
UPE regulated the balance of Th1/Th2/Th17/Treg‐associated cytokines in NALF. Th1 (A, B) and Treg (C)‐related cytokines were upregulated in NALF, while Th2 (D–F) and Th17 (G)‐related cytokines were downregulated. Data were obtained from the results of three separate experiments and displayed as mean ± *SD*. ANOVA test was applied to compare the differences among multiple groups, respectively. Significant differences at ##*p* < .01, ###*p* < .001 compared with the control group. Significant differences at *p < .05, **p < .01, and ***p < .001 compared with the OVA group. ANOVA, analysis of variance; NALF, nasal lavage fluid; OVA, ovalbumin; UPE, *Undaria pinnatifida* extract.

In this study, we conducted an analysis of Th2 (IL‐4, IL‐13), Th1 (IFN‐γ, IL‐12), Th17 (IL‐17), and Treg (IL‐10) cytokine concentrations in NALF. The objective was to analyze the impact of UPE on the modulation of T helper cell reactions. The concentrations of IL‐4, IL‐13, and IL‐17 in the NALF of the group exposed to OVA were found to be significantly elevated compared to the control group. Conversely, the levels of IL‐12, IFN‐γ, and IL‐10 in the OVA group were significantly lower than those observed in the control group (Figure 7A−G). The oral administration of UPE significantly downregulated the IL‐4, IL‐13, and IL‐17 levels in a dose‐dependent manner. In addition, non‐significant upregulation of Th1 cytokines INF‐γ, IL‐12, and IL‐10 in Tregs was observed in UPE‐treated mice in NALF (Figure 7A−G). Similarly, Dex‐treated mice exhibited significant decrease in IL‐4, IL‐13, and IL‐17, and increased IFN‐γ, IL‐12, and IL‐10 levels compared with the OVA group. These results suggest that the oral administration of UPE can reverse abnormal allergic immune responses by enhancing Th1 and Treg, inhibiting Th2 and Th17, and modulating the balance of Th1/Th2/Th17/Treg.

### 3.6 UPE inhibited the infiltration of eosinophils in the nasal mucosa

**Figure 8:**
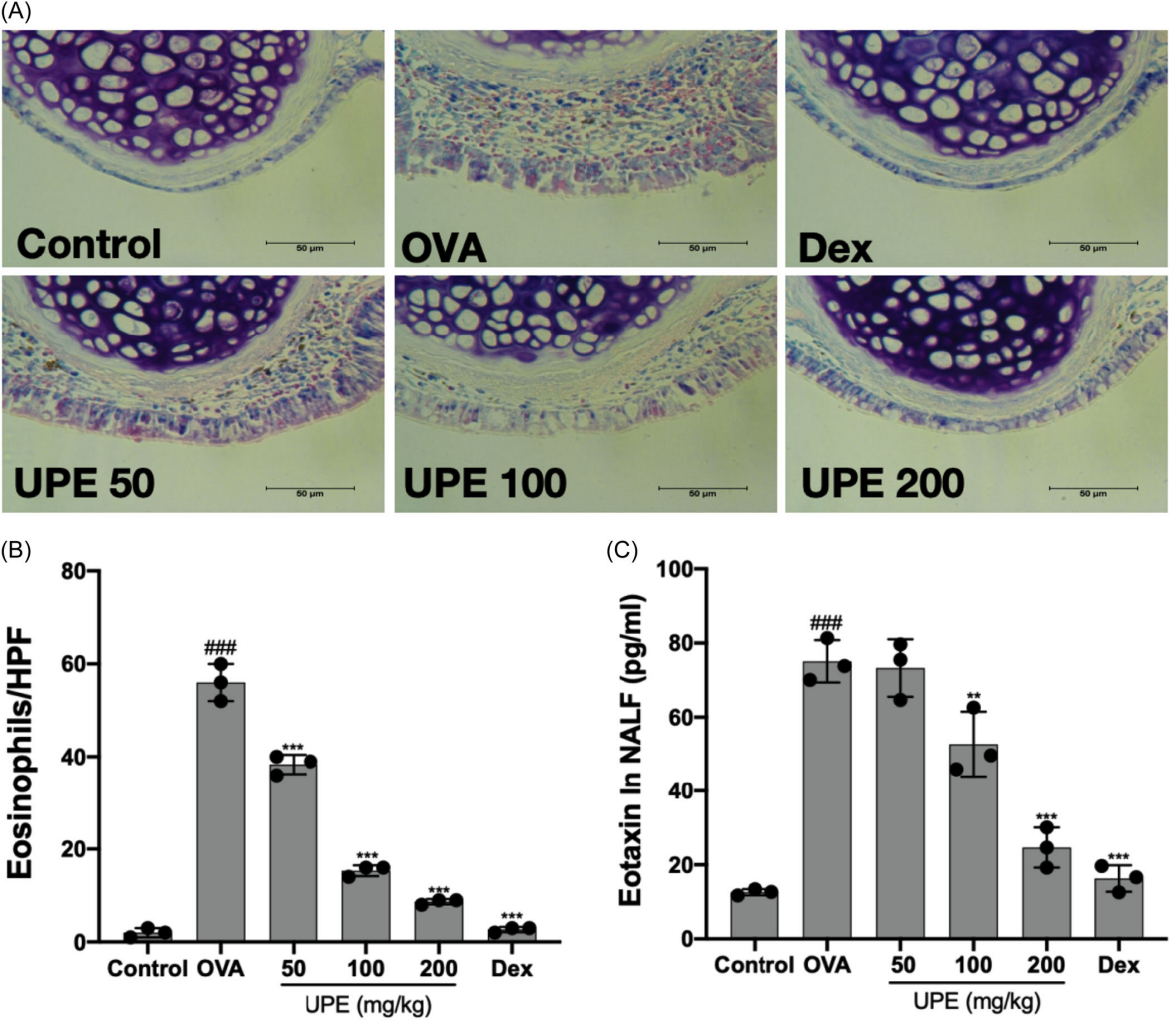
UPE inhibited the accumulation and the activation of eosinophils in the nasal mucosa. (A) Eosinophils were identified by Giemsa staining (B) The number of eosinophils in the nasal mucosa was counted under a high magnification field of view (C) The level of eotaxin was measured in NALF. Scale bar 50 μm, ×400 magnification. Data were obtained from the results of three separate experiments and displayed as mean ± *SD*. ANOVA test was applied to compare the differences among multiple groups, respectively. Significant differences at ###*p* < .001 compared with the control group. Significant differences at **p < .01, ***p < .001 compared with the OVA group. ANOVA, analysis of variance; NALF, nasal lavage fluid; OVA, ovalbumin; UPE, *Undaria pinnatifida* extract.

Following an allergic reaction, there is a notable accumulation of eosinophils inside the nasal mucosa. These eosinophils then release various inflammatory mediators, which in turn induce inflammation within the airways. Using Giemsa staining on histopathological sections showed that eosinophil cytoplasm turned red, which means that there were too many eosinophils in the OVA group. However, the administration of UPE and Dex effectively suppressed eosinophil infiltration in the nasal mucosa (Figure 8A). In AR mice, the presence of sub‐epithelial eosinophils was significantly higher compared with that in the control mice; after UPE and Dex treatments, the presence of eosinophils was significantly reduced in a dose‐dependent manner (Figure 8B). Eosinophils are known to be overproduced in response to the eosinophil chemotactic protein CCL11.^30^ Eotaxin was found to be significantly elevated in the NALF of OVA‐exposed mice, and inhibited in a dose‐dependent manner by UPE treatment (Figure 8C).

### 3.7 UPE inhibited the NF‐κB/MAPKs signaling pathway in lung tissue

**Figure 9:**
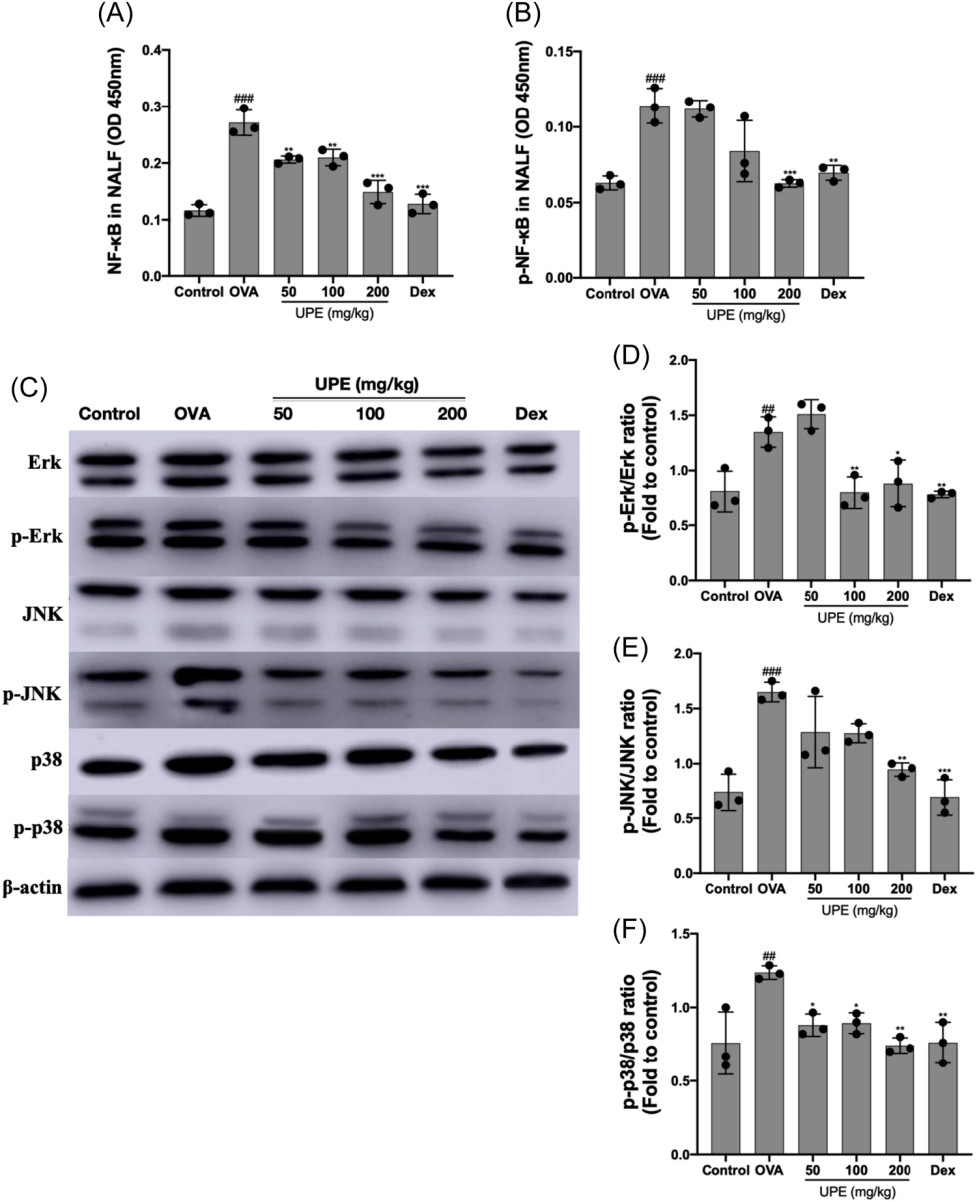
UPE inhibited the NF‐κB/MAPKs signaling pathway in NALF and lung. The transcription factor NF‐κB in NALF was detected by ELISA kit, and the MAPKs were measured by western blot analysis in the lung tissue. (A, B) NF‐κB, phosphorylation levels of NF‐κB in NALF (C) Western blot analysis of MAPKs (D–F) p‐Erk/Erk ratio, p‐JNK/JNK ratio, p‐p38/p38 ratio. Data were obtained from the results of three separate experiments and displayed as mean ± *SD*. ANOVA test was applied to compare the differences among multiple groups, respectively. Significant differences at ##*p* < .01, ###*p* < .001 compared to the control group. Significant differences at *p < .05, **p < .01, ***p < .001 compared with the OVA group. ANOVA, analysis of variance; MAPK, mitogen‐activated protein kinases; NALF, nasal lavage fluid; NF‐κB, nuclear factor kappa B; OVA, ovalbumin; UPE, *Undaria pinnatifida* extract.

NF‐κB is a transcription factor that plays a central role in the regulation of inflammatory responses.^31^ MAPKs, such as c‐Jun N‐terminal kinase (JNK), extracellular signal‐regulated kinase (Erk), and p38MAPK, are thought to be critical regulators of inflammation.^32^ As shown in Figure 9A−F, OVA‐challenged mice contained significantly elevated phosphorylation of MAPKs in the lung tissue, total NF‐κB, and phosphorylation of NF‐κB in NALF compared with that of control mice; however, significant inhibition was noticed in Dex and UPE‐treated mice compared with the OVA‐challenged mice.

### 3.8 UPE inhibited the compound 48/80‐induced RPMCs degranulation

**Figure 10:**
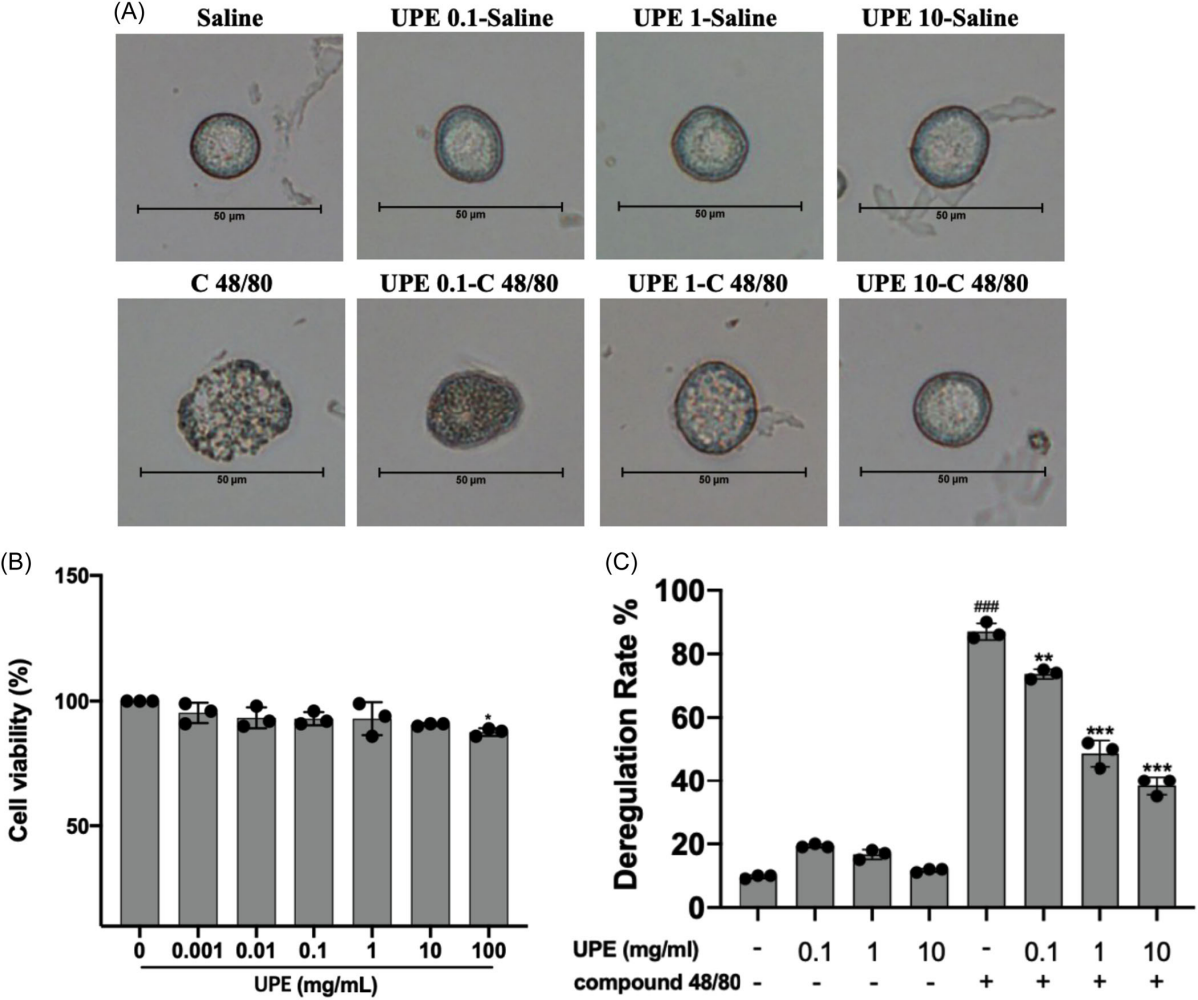
UPE suppressed the compound 48/80‐induced mast cell degranulation. (A) Morphologies of C48/80‐stimulated rat peritoneal mast cells pretreated with UPE. (B) Cytotoxicity of UPE in RPMCs. (C) Mast cell degranulation rates. Data were obtained from the results of three separate experiments and displayed as mean ± *SD*. ANOVA test was applied to compare the differences among multiple groups, respectively. Scale bar 50 μm, ×400 magnification. Significant differences at ###*p* < .001 compared with the non‐UPE and C48/80 groups. Significant differences at *p < .05 compared with the UPE (0 mg/mL) group. **p < .01, ***p < .001 compared with the C48/80 group. ANOVA, analysis of variance; UPE, *Undaria pinnatifida* extract.

In this study, RPMCs were used to evaluate the in vitro anti‐allergic and inflammatory effects of UPE. RPMCs were treated with C48/80, and the morphological changes were observed. Extensive degranulation was evident in treated RPMCs (Figure 10A); however, degranulation was inhibited, and the morphology was similar to that of saline‐treated RPMCs when RPMCs were treated with UPE (at doses of 0.1, 1, and 10 mg/mL) 15 min before stimulation with C48/80. RPMCs were treated with various concentrations of UPE, and we found no significant cell toxicity at 10 mg/mL (Figure 10B). Figure 10C showed C48/80 induced RPMCs degranulation rates when these cells were untreated and pretreated with UPE.

### 3.9 UPE inhibited the infiltration of mast cells and attenuated the release of histamine

**Figure 11:**
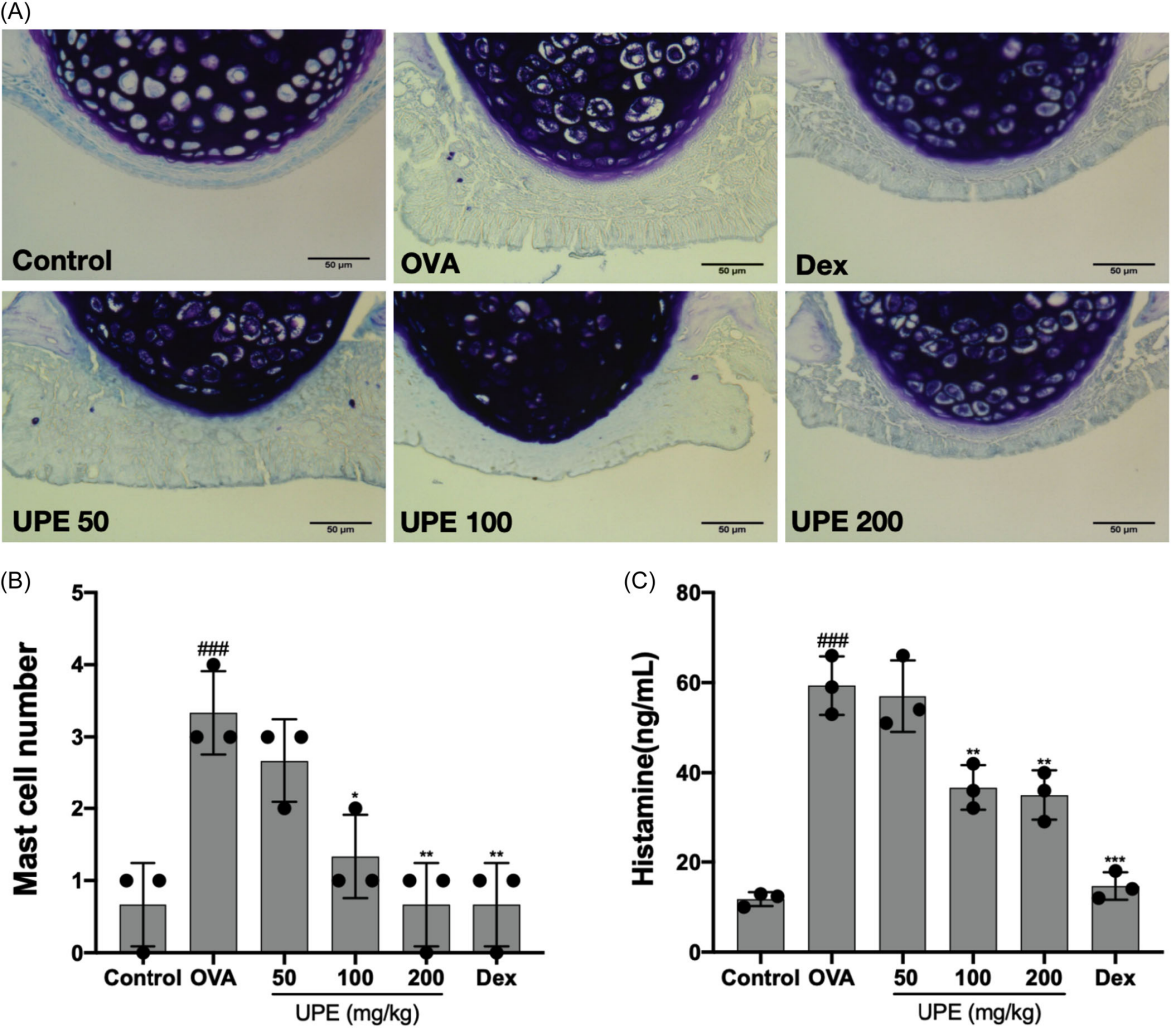
UPE inhibited the infiltration of mast cells and the secretion of histamine in NALF. (A) Mast cells were identified by Toluidine Blue staining. (B) The number of mast cells in the nasal mucosa. (C) The levels of histamine were measured in NALF. Data were obtained from the results of three separate experiments and displayed as mean ± *SD*. ANOVA test was applied to compare the differences among multiple groups, respectively. Scale bar 50 μm, ×400 magnification. Significant differences at ###*p* < .001 compared with the control group. Significant differences at *p < .05, **p < .01, ***p < .001 compared with the OVA group. ANOVA, analysis of variance; NALF, nasal lavage fluid; OVA, ovalbumin; UPE, *Undaria pinnatifida* extract.

During the AR reaction, antibodies exhibit a special affinity for mast cells located in the nasal mucosa, resulting in the clustering of mast cells and the subsequent release of histamine and proinflammatory cytokines.^33^ There was a statistically significant increase in the quantity of mast cells observed in the nasal mucosa of the OVA group compared to the control group (Figure 11A,B). Administration of UPE and Dex suppressed mast cell infiltration into the nasal mucosa. The secretion of histamine in NALF was also analyzed; histamine level was upregulated in mice exposed to OVA and downregulated in a dose‐dependent manner after UPE treatment (Figure 11C).

## 4 DISCUSSION

The objective of this work was to examine the potential anti‐allergic and anti‐inflammatory properties of UPE through the utilization of an OVA‐induced AR mice model. The present investigation aimed to assess the scores of rubbing and sneezing symptoms during a 15‐min timeframe after the final nasal challenge with OVA. The nasal allergy symptoms were greatly suppressed with the oral intake of UPE. OVA‐challenged mice showed increased numbers of inflammatory cells in the NALF, elevated serum OVA‐specific IgE and IgG_1_, and decreased IgG_2a_. The Th2 cytokines, namely IL‐4, IL‐5, and IL‐13, have been documented to augment the synthesis of IgE and govern the proliferation and specialization of eosinophils.^34^ On the other hand, Th1 cells produce IL‐12 and IFN‐γ, which play a crucial role in suppressing the manufacture of IgE and preventing the development of IgE‐mediated allergic reactions.^35^ Th17 cells, a subset of CD4+ T‐cells, are associated with a more severe asthma phenotype,^36^ and IL‐17 can increase NF‐κB activation and secretion of neutrophil chemokines.^37^ Tregs play a central role in regulating autoimmune, infectious, and allergic diseases by cell‐to‐cell contact‐dependent inhibition and secretion of anti‐inflammatory cytokines such as IL‐10 and TGF‐β.^38^ Based on these factors, the study shows that UPE might be able to stop inflammatory cells from activating by controlling the balance between Th1, Th2, Th17, and Treg cells.

The involvement of antigen presentation cells and T and B lymphocytes in the early stages of AR is widely recognized. This process results in the activation of mast cells upon re‐exposure to the specific allergen, which is primarily mediated via the cross‐linking of the high‐affinity receptor for IgE antibodies (FcεRI) on the cell membrane. This mechanism is well documented and understood.^39^ IgE cross‐linking on mast cells leads to the release of allergic mediators (e.g., histamine, TNF‐α, and IL‐6) and results in immediate nasal symptoms.^40^ Depending on the receptor expression pattern in T‐cells, histamine can induce the production of Th2‐specific cytokines, such as IL‐4 and IL‐13.^41^ In the present study, the mast cell and mast cell degranulation numbers, observed in nasal histological analyses, decreased after UPE treatment in comparison to the OVA group. According to these results, the release of histamine was also significantly inhibited. Moreover, some studies have shown that mast cells are necessary for an enhanced influx of eosinophils into the lung^42^ for the induction of increased airway hyperresponsiveness, or the induction of subepithelial fibrosis.^43^ Eosinophils infiltrate the nasal cavity in the late response,^44^ and eosinophil‐derived mediators can induce epithelial cell damage, leading to swelling of the nasal mucosa.^45^ Furthermore, the Th2 cytokine IL‐5 can activate signaling networks in eosinophils, including NF‐κB and MAPKs, and the combined stimulation of these kinases and transcription factors drives eosinophil differentiation, survival, degranulation, and recruitment.^46^ Typically, the NF‐κB complex forms an association with IκB molecules within the cytoplasm, resulting in a state of inactivity. When pathogens are activated, the NF‐κB signaling pathway is set off. This causes IκBα to break down and NF‐κB p65 and p50 dimers to form.^47^ Following translocation to the nucleus, NF‐κB p65 undergo translation, leading to the synthesis of inflammatory mediators, including IL‐6 and TNF‐α, as well as Th2 cytokines (IL‐4, IL‐5, and IL‐13) in the context of allergic airway inflammation.^48^ Upon activation of pathogens via IgE‐mediated responses, MAPKs, including p38, ERK, and JNK, are phosphorylated. The nuclear translocation of AP‐1, which transcribes and generates cytokines, is facilitated by these MAPKs. Moreover, phosphorylation of MAPKs (p38 MAPK, Erk, and JNK) can regulate a variety of cellular activities, including gene expression, differentiation, and differentiation in response to external stimuli.^49^ Anti‐MAPK inhibitors have been manufactured with the intention of managing a wide range of inflammatory disorders.^50^ In this study, UPE promotes the stability of eosinophils and mast cells. By inhibiting NF‐κB and MAPKs and suppressing nuclear transcription factors, this mechanism is carried out. Thus, UPE can protect the nasal cavity from mucosal swelling, eosinophilia, and mucus hyperplasia in an OVA‐induced AR mouse model.

## 5 CONCLUSION

Aral UPE attenuated allergic reactions by inhibiting mast cell aggregation, decreasing serum OVA‐specific IgE and IgG_1_ expression, and increasing IgG_2a_ expression. UPE improved nasal inflammation by reducing eosinophil infiltration and other inflammatory cells such as neutrophils, macrophages, and goblet cells in the nasal mucosa. Furthermore, under UPE treatment, a dynamic balance was established between Th1/Th2/Th17/Treg‐related cytokines via inhibiting NF‐κB/MAPKs signaling pathway, thus preventing nasal inflammation and eosinophil activation. The results obtained in this study suggest that UPE may be a promising strategy for immunotherapy.

## AUTHOR CONTRIBUTIONS

Zhen Nan Yu, and Ok Hee Chai conceived and designed this study. Zhen Nan Yu, Yan Jing Fan, Thi Van Nguyen, and Chun Hua Piao performed the experiments. Zhen Nan Yu, Hee Soon Shin, Byung‐Hoo Lee, So‐Young Lee Chang Ho Song, and Ok Hee Chai analyzed and interpreted the data. Zhen Nan Yu, and Ok Hee Chai wrote and edited the manuscript, which was approved by all authors. All authors have read and agreed to the submission of the manuscript.

## CONFLICT OF INTEREST STATEMENT

The authors declare no conflicts of interest.

## Data Availability

The data presented in this study are available upon request from the corresponding author.
